# Identification of T-Cell Antigens Specific for Latent *Mycobacterium Tuberculosis* Infection

**DOI:** 10.1371/journal.pone.0005590

**Published:** 2009-05-18

**Authors:** Sebastian D. Schuck, Henrik Mueller, Frank Kunitz, Albert Neher, Harald Hoffmann, Kees L. C. M. Franken, Dirk Repsilber, Tom H. M. Ottenhoff, Stefan H. E. Kaufmann, Marc Jacobsen

**Affiliations:** 1 Department of Immunology, Max Planck Institute for Infection Biology, Berlin, Germany; 2 Respiratory Diseases Clinic Heckeshorn, Department of Pneumology, HELIOS Klinikum Emil von Behring, Berlin, Germany; 3 Asklepios Professional Clinic München-Gauting, Centre for Pneumology and Thorax Surgery, Munich, Germany; 4 Department of Immunohematology & Blood Transfusion/Department of Infectious Diseases, Leiden University Medical Center, Leiden, The Netherlands; 5 Research Institute for the Biology of Farm Animals, Genetics and Biometry, Dummerstorf, Germany; New York University School of Medicine, United States of America

## Abstract

**Background:**

T-cell responses against dormancy-, resuscitation-, and reactivation-associated antigens of *Mycobacterium tuberculosis* are candidate biomarkers of latent infection in humans.

**Methodology/Principal Findings:**

We established an assay based on two rounds of *in vitro* restimulation and intracellular cytokine analysis that detects T-cell responses to antigens expressed during latent *M. tuberculosis* infection. Comparison between active pulmonary tuberculosis (TB) patients and healthy latently *M. tuberculosis*-infected donors (LTBI) revealed significantly higher T-cell responses against 7 of 35 tested *M. tuberculosis* latency-associated antigens in LTBI. Notably, T cells specific for Rv3407 were exclusively detected in LTBI but not in TB patients. The T-cell IFNγ response against Rv3407 in individual donors was the most influential factor in discrimination analysis that classified TB patients and LTBI with 83% accuracy using cross-validation. Rv3407 peptide pool stimulations revealed distinct candidate epitopes in four LTBI.

**Conclusions:**

Our findings further support the hypothesis that the latency-associated antigens can be exploited as biomarkers for LTBI.

## Introduction

In the vast majority of individuals, specific cellular immunity against *M. tuberculosis* is capable of controlling infection leading to latent *M. tuberculosis* infection (LTBI) [1]. LTBI is thought to be associated with a dormancy/non-replicating state of low metabolic activity of the pathogen. Dormancy-related (DosR) antigens as well as proteins expressed during reactivation and resuscitation of dormant bacilli are candidate biomarkers for LTBI and disease reactivation [2], [3], [4]. A limited number of studies tested latency-associated antigens in immunologic assays. These showed that DosR antigens induced T-cell cytokine expression in humans [5] and mice [6]. Resuscitation-promoting factors (rpf) induced immune responses in mice [7] but have not been tested in humans. Recent studies based on *M. tuberculosis* knock-out strains revealed that Rv3407, a protein which is not expressed in *M. bovis* BCG at detectable abundance [8], is under the control of two rpf [9]. Neither Rv3407 nor reactivation-associated antigens (i.e. Rv0104, Rv1115 [10]) have been tested for immunogenicity so far. For reasons of comprehensibility we use the term latency-associated antigens for *M. tuberculosis* proteins involved in dormancy, resuscitation, and reactivation of *M. tuberculosis*.

We tested the immunogenicity of 35 latency-associated antigens using different assays based on intracellular cytokine staining for IFNγ and IFNγ-ELISA. IFNγ is a crucial mediator of protection against tuberculosis which strongly depends on T helper type-1 immunity. IFNγ activates infected macrophages at the site of bacterial residence - an essential mechanism for the killing of mycobacteria [11]. IFNγ release in response to immunodominant antigens (i.e. ESAT6, CFP10) is used in standard tests for *M. tuberculosis* infection (IFNγ release assays, IGRA) [12].

IGRA as well as standard intracellular cytokine staining methods are based on short-term incubation between 6 and 24 h [13]. Principally short-term assays detect recent *M. tuberculosis* infection while prolonged *in vitro* stimulation increases sensitivity for LTBI [14], [15], [16]. Yet, these assays remain insufficient as robust correlates of protection against *M. tuberculosis* [17]. Therefore, biomarkers which reliably predict protective immunity are urgently needed [18]. Antigens predominantly expressed by dormant *M. tuberculosis* during LTBI are promising candidate immune markers of protection [5]. We therefore decided to develop an assay based on two rounds of *in vitro* restimulation, to determine IFNγ production in response to *M. tuberculosis* latency-associated antigens in LTBI, TB patients, and tuberculin skin test (TST)-negative donors. Differentially expressed proteins were then tested for their capacity to discriminate between LTBI and TB patients. Finally, overlapping peptide pools were applied to identify immunogenic epitopes of the most promising candidate, Rv3407.

## Results

### Two-rounds of in vitro restimulation detect T-cell responses against latency-associated antigens

**Figure 1:**
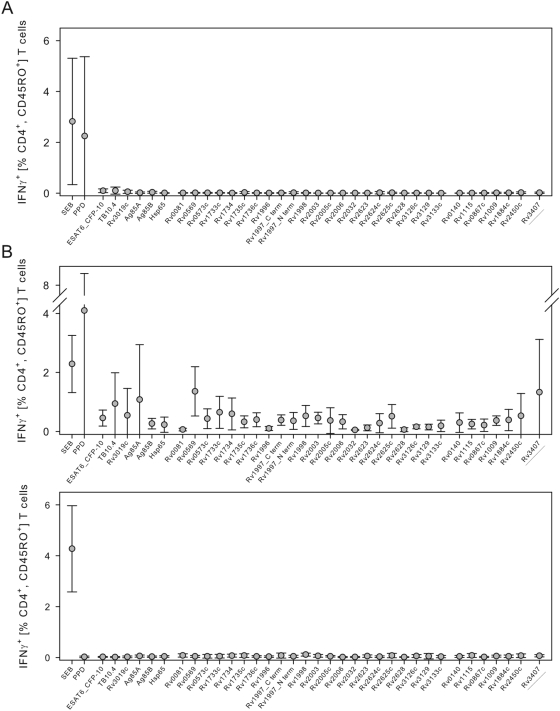
IFNγ-expressing CD4^+^ CD45RO^+^ T cells after 16 h and 7 days restimulation with immunodominant and latency-associated antigens from LTBI and TST-negative controls. Intracellular cytokine expression after 16 h restimulation in PBMC from LTBI (A), and 7 days – including two rounds of *in vitro* restimulation – in PBMC from LTBI (B, upper graph) and TST-negative donors (B, lower graph) are shown. Scatter plots indicate mean and standard deviation. Percentages of IFNγ-expressing CD4^+^ CD45RO^+^ memory T cells are indicated on the y-axis for SEB, PPD from *M. tuberculosis*, and tested antigens (x-axes). Background values of non-stimulated controls were subtracted for each individual donor. The most promising candidate Rv3407 is underlined. PPD: purified protein derivative of *M. tuberculosis*; SEB: Staphylococcus enterotoxin B.

We assessed 29 antigens associated with *M. tuberculosis* dormancy, resuscitation, and reactivation (in short latency, Table 1) for their potential to elicit recall responses after 16 h short-term *in vitro* stimulation of PBMC from LTBI. We detected IFNγ expression in CD4^+^CD45RO^+^ memory T cells from LTBI after stimulation with PPD from *M. tuberculosis* and, to a minor degree, with *M. tuberculosis*-immunodominant proteins ESAT6_CFP-10, TB10.4, and Ag85a (figure 1*A*). Notably, none of the latency-associated antigens induced detectable cytokine expression after 16 h in LTBI (figure 1A) and no T-cell responses against *M. tuberculosis*-specific antigens were detected in 10 tuberculin skin test (TST)-negative donors (data not shown).

**Table 1:** List of proteins candidates.

Name	Category
ESAT6_CFP-10	Immunodominant *M. tuberculosis* proteins
TB10.4	
Rv3019c	
Ag85A	
Ag85B	
Hsp65	
Rv0081	DosR* regulon-encoded M. tuberculosis proteins [2]
Rv0569	
Rv0573c	
Rv1733c	
Rv1734	
Rv1735c	
Rv1736c	
Rv1996	
Rv1997_C term	
Rv1997_N term	
Rv1998	
Rv2003	
Rv2005c	
Rv2006	
Rv2032	
Rv2623	
Rv2624c	
Rv2625c	
Rv2628	
Rv3126c	
Rv3129	
Rv3133c	
Rv0140	*M. tuberculosis* reactivation-associated proteins [10]
Rv1115	
Rv0867c	*M. tuberculosis* resuscitation promoting factors [30]
Rv1009	
Rv1884c	
Rv2450c	
Rv3407	*M. tuberculosis* resuscitation-associated protein [19]

* *M. tuberculosis* dormancy-related antigens.

Increased T cell-derived IFNγ responses after prolonged *in vitro* incubation have been described [5], [14], [16]. Since our own previous experiments indicated that a second stimulation before measurement with the same antigen is optimal for intracellular cytokine detection (data not shown), we combined 7 days of stimulation with restimulation 16 h prior to analysis. Frequencies of IFNγ-expressing T cells in PBMC from LTBI were markedly increased after stimulation with immunodominant proteins or PPD for 7 days (figure 1B, upper graph). In contrast to the short-term single stimulation assay, latency-associated antigens induced IFNγ expression in memory T cells from the majority of LTBI in the long-term restimulation assay (figure 1B, upper graph). Two of these latency-associated antigens, namely Rv0569 and Rv3407, induced IFNγ expression comparable to the ESAT6_CFP-10 fusion protein, the immunodominant antigen which induced the highest frequencies of cytokine-expressing cells in LTBI. In contrast, none of the antigens induced detectable IFNγ expression in T cells from TST-negative donors in this assay (figure 1B, lower graph). Hence, the “7-day two rounds of restimulation” assay detects specific T-cell responses in LTBI which are missed by the 16h short-term assay.

Comparisons for the individual donor T-cell responses after 7 days and two rounds of restimulation revealed comparable results against three selected immunodominant antigens (i.e. ESAT6_CFP-10, TB10.4, Ag85A) and three latency-associated antigens (i.e. Rv0569, Rv1734, Rv2003) (data not shown). Considering significantly higher proportions of T cells specific for immunodominant antigens in the short-term assay, a simply ‘boosted’ T-cell response by prolonged stimulation would not explain equal proportions since this would have led to higher proportions of T cells specific for immunodominant antigens in the 7-day assay.

**Figure 2:**
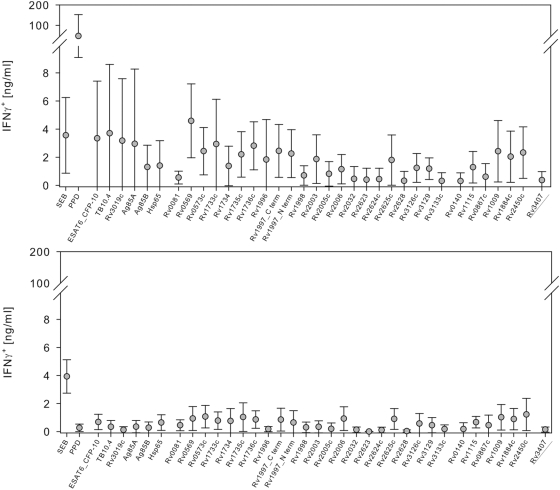
IFNγ ELISA analyses after restimulation with immunodominant and latency-associated antigens of PBMC from LTBI and TST-negative donors. Analyses of IFNγ in the culture supernatant by ELISA after 7 days and two rounds of *in vitro* restimulation in PBMC from LTBI (*A*) and TST-negative donors (*B*) are shown. Scatter plots indicate mean and standard deviation. Background values of non-stimulated controls were subtracted. IFNγ concentrations in the supernatant are indicated on the y-axis for stimulation with SEB, PPD from *M. tuberculosis*, and tested antigens (x-axes). The most promising candidate Rv3407 is underlined.

Analyses of IFNγ in supernatants by ELISA at day 7 following two rounds of restimulation revealed a similar tendency for PPD, immunodominant, and latency-associated *M. tuberculosis* proteins in LTBI (figure 2A). In contrast to intracellular staining, a considerable amount of IFNγ was also detected in supernatants of PBMC from some TST-negative donors after stimulation with latency-associated antigens (figure 2B). Therefore intracellular measurement of IFNγ after two rounds of *in vitro* restimulation revealed higher specificity as compared to the IFNγ ELISA.

### Significantly stronger T-cell responses in LTBI as compared to TB patients against latency-associated antigens

**Figure 3:**
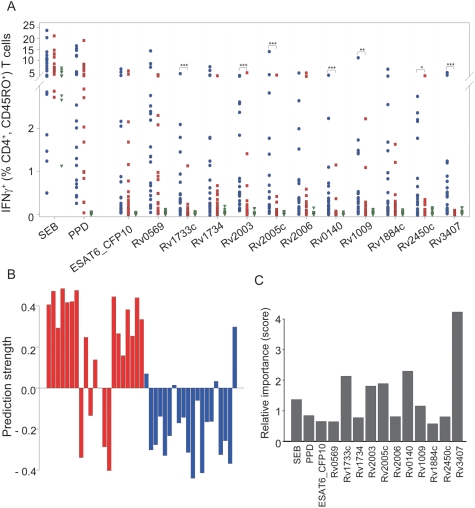
Comparison of IFNγ-expressing CD4^+^ T cells specific for immunodominant and latency-associated M. tuberculosis antigens between patients with TB, LTBI, and TST-negative donors. (A). Percentages of IFNγ-expressing CD4^+^ CD45RO^+^ memory T cells are shown for stimulation with SEB, PPD from *M. tuberculosis*, and 11 latency-associated antigens after 7 days and two rounds of *in vitro* restimulation. T-cell responses from TST-negative donors are indicated as green circles, LTBI are indicated as blue squares, and TB patients are indicated as red triangles. Two-sided p-values for the Mann-Whitney U-test are indicated as follows: * *P*<0.05, ** *P*<0.01; and *** *P*<0.001. (B) Classification of TB patients and LTBI based on random forest analysis using 11 latency-associated antigens as well as ESAT6_CFP-10, and PPD. Results from the cross validation are shown in a bar chart. Each bar represents an individual donor. TB patients are shown on the left (red bars), LTBI on the right side (blue bars). The y-axis indicates the prediction threshold calculated by random forest analysis. Negative bars predict a TB patient, positive bars an LTBI. The prediction probability is represented as the bar height. (C) Mean decrease of class impurity over all trees measured as Gini index (y-axis) indicates the relative importance of each factor (x-axis) for classification. PPD: purified protein derivative of *M. tuberculosis*; SEB Staphylococcus enterotoxin B.

The expression of the vast majority of antigens used in this study has been associated with latent stages of *M. tuberculosis* infection. Consequently, we addressed the question whether these antigens are differentially recognized by T cells from patients with active pulmonary TB and LTBI. Eleven latency-associated antigens (i.e. Rv0569, Rv1733c, Rv1734, Rv2003, Rv2005c, Rv2006, Rv0140, Rv1009, Rv1884c, Rv2450c, and Rv3407), which induced strong responses in LTBI, were selected for these experiments. Seven of these latency-associated antigens induced significantly higher T-cell responses in LTBI as compared to TB patients (*P*<0.001 for Rv1733c, Rv2003, Rv2005c, Rv0140, and Rv3407; *P*<0.01 for Rv1009; *P*<0.05 for Rv2450c) (figure 3A).

We set a threshold of 0.2% IFNγ^+^, CD4^+^, CD45RO^+^ T cells (i.e. 20-fold above the assumed flow cytometric detection threshold of 0.01%) to define positive T-cell responses against latency-associated antigens in LTBI and TB patients for the most promising candidates (*P*<0.001) (Table 2). Latency-associated antigens induced T-cell responses in the range of 45.5 to 72.7% of LTBI patients. A specific T-cell response against Rv2003 was detected in the vast majority of LTBI (16 of 22) but also induced positive responses in 25% (5 of 20) of the TB patients. Notably, the resuscitation-associated antigen Rv3407 [19] induced positive T-cell responses in 12 LTBI (55%) but in none of the TB patients. These results prompted us to determine whether T-cell responses against *M. tuberculosis* antigens are sufficient for classification of TB patients and LTBI.

**Table 2:** Positive T-cell responses against latency-associated antigens.

Antigen	LTBI^1^(%)	TB patients^1^(%)
Rv2003	16 (72.7)	5 (25)
Rv1733c	15 (68.2)	2 (10)
Rv3407	12 (54.5)	0 (0)
Rv2005c	12 (54.5)	3 (15)
Rv0140	10 (45.5)	2 (10)

1 Number of donors with more than 0.2% IFNγ-producing T cells.

### T-cell responses against latency-associated antigens discriminate between LTBI and TB patients

We included the 11 immunogenic latency-associated antigens together with ESAT6_CFP-10, and PPD in a discrimination approach. Random forest analyses together with leave-1-out cross-validations across all possible combinations were applied to determine the prediction accuracy and relative importance of each factor for classification. These analyses revealed a cross-validated prediction accuracy of 83% between TB patients and LTBI (figure 3B). The relative feature importance for discrimination showed that Rv3407 was by far the most influential factor for classification (figure 3C). Consequently we designed overlapping peptide pools of Rv3407 to identify immunogenic epitopes.

### Identification of immunogenic peptides of Rv3407

**Figure 4:**
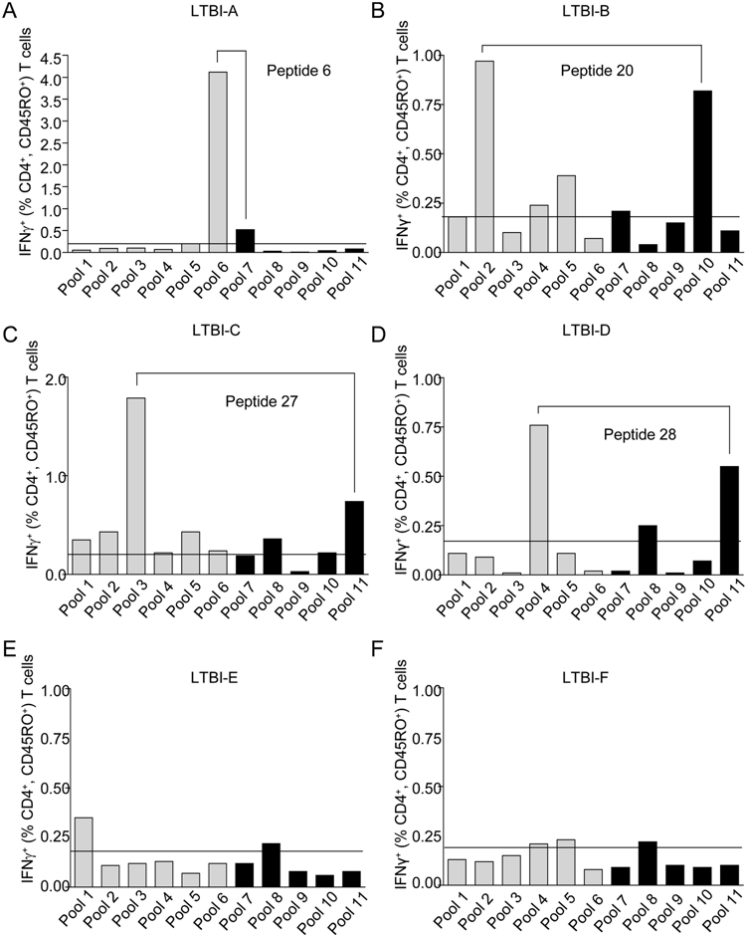
Overlapping peptide pools of latency-associated protein Rv3407 stimulate IFNγ-expressing CD4^+^ T cells after 7 days and two rounds of restimulation. PBMC from six LTBI (A–F) were restimulated with 15-mer synthetic peptide pools of Rv3407 for 7 days including two rounds of *in vitro* restimulation. IFNγ-expressing CD4^+^ CD45RO^+^ T cells are shown for stimulation with peptide pools 1 to 6 (grey bars) and pools 7 to 11 (black bars). Each peptide is constituent of one pool within pools 1 to 6 and of one pool within pools 7 to 11. Peptides inducing the most prominent responses are indicated for donors *A–D*. The horizontal line indicates the threshold for positive responses (0.2%). Background values of non-stimulated controls were subtracted.

The matrix design of the overlapping peptide pools leads to six ‘vertical’ and five ‘horizontal’ pools. Each of the 29 peptides of Rv3407 was present in two pools (Table 3). In 6 out of 10 LTBI tested – those with the highest T-cell response against Rv3407 – a minimum of 0.2% IFNγ-expressing CD4^+^ T cells was detected after stimulation with at least two of the peptide pools (figure 4). In four LTBI (LTBI-A to LTBI-D) a major immunogenic epitope could be identified (figure 4A–D) while in two LTBI (LTBI-E, LTBI-F) with the weakest response against the Rv3407 protein no prominent epitope-specific T-cell responses were observed (figure 4E–F). Notably peptide 6 (LRQHASRYLARVEAG) identified in LTBI-A induced IFNγ expression in about 1% of CD8^+^ memory T cells, as well (data not shown). Therefore distinct peptide epitopes in different LTBI were recognized by antigen-specific T cells and ongoing studies will determine whether this is due to differential HLA-phenotypes.

**Table 3:** Design of Rv3407 peptide pools.

No.^1^	Amino acid sequence
P1	MRATVGLVEAIGIRE
P2	TVGLVEAIGIRELRQ
P3	LVEAIGIRELRQHAS
P4	AIGIRELRQHASRYL
P5	IRELRQHASRYLARV
P6	LRQHASRYLARVEAG
P7	HASRYLARVEAGEEL
P8	RYLARVEAGEELGVT
P9	ARVEAGEELGVTNKG
P10	EAGEELGVTNKGRLV
P11	EELGVTNKGRLVARL
P12	GVTNKGRLVARLIPV
P13	NKGRLVARLIPVQAA
P14	RLVARLIPVQAAERS
P15	ARLIPVQAAERSREA
P16	IPVQAAERSREALIE
P17	QAAERSREALIESGV
P18	ERSREALIESGVLIP
P19	REALIESGVLIPARR
P20	LIESGVLIPARRPQN
P21	SGVLIPARRPQNLLD
P22	LIPARRPQNLLDVTA
P23	ARRPQNLLDVTAEPA
P24	PQNLLDVTAEPARGR
P25	LLDVTAEPARGRKRT
P26	VTAEPARGRKRTLSD
P27	EPARGRKRTLSDVLN
P28	RGRKRTLSDVLNEMR
P29	KRTLSDVLNEMRDEQ

1 Peptide number according to the position within the primary sequence from C- to N-terminus.

We consider the detection of specific T-cell immunity against latency-associated antigens and the identification of immunogenic epitopes an initial step towards definition of reliable biomarkers for protective immunity against TB.

## Discussion

T-cell immunity in LTBI is crucial for protection against resuscitation and reactivation of *M. tuberculosis*. We established two rounds of *in vitro* restimulation assay that induced specific T-cell immunity against latency-associated antigens which were not detected in a short-term T-cell assay. These intracellular IFNγ responses were significantly increased in LTBI as compared to TB patients. This assay was highly specific since IFNγ-expressing memory T cells were only detected in PBMC from LTBI and not from TST-negative donors.

We identified a subgroup of latency-associated antigens that induced significantly stronger T-cell responses in LTBI as compared to TB patients. This is in accordance with a previous study that showed increased responses against the latency-associated antigen Rv1733c in LTBI as compared to TB patients [5]. The remaining candidates (i.e. DosR regulon-encoded proteins Rv2003 and Rv2005c, the reactivation-associated protein Rv0140, the resuscitation promoting factors Rv2450c and Rv1009, as well as Rv3407) have not been tested so far. Rv3407 induced IFNγ in T cells exclusively of LTBI. We identified Rv3407 previously by comparative proteomics of *M. tuberculosis* strain H37Rv and *M. bovis* BCG [8], [19]. Rv3407 improved the vaccine efficacy of BCG suggesting that subunit vaccination can be used to improve pre-existing protection evoked by BCG [19]. Ongoing studies in our institute aim at characterizing the biological and antigenic role of Rv3407. Furthermore all immunogenic protein candidates identified in this study will be analyzed for the induction of protective T-cell responses in TB high-incidence countries (www.biomarkers-for-tb.net).

T-cell responses against this subgroup of latency-associated antigens classified LTBI and TB patients with an accuracy of 83% and Rv3407 contributed significantly to discrimination. The discrimination efficacy was comparable to studies of blood cell counts and *ex vivo* serum cytokine analyses in fast and slow treatment responders [20] and was only slightly lower than RNA-based candidate markers in PBMC from TB patients and LTBI [21] or a serum-based proteomic approach [22].

We determined IFNγ expression in T cells specific for latency-associated antigens. TNFα-expression revealed comparable results and a strongly overlapping cytokine expression pattern (data not shown) while IL-2 expression was not detectable after 7 days of *in vitro* restimulation (data not shown). Therefore T cells induced by latency-associated antigens show a phenotype of multifunctional T cells, which have been shown to protect against TB in the mouse model [23]. We have shown recently that GM-CSF expressing multifunctional T cells are not increased in TB patients while other cytokines were increased in TB patients as compared to LTBI [24]. Ongoing longitudinal prospective studies analyze whether the frequency and phenotype of latency antigen-specific T cells in LTBI is associated with the risk of developing TB at a later time point and hence can be exploited for prognostic diagnosis (www.biomarkers-for-tb.net). One important aspect that will be addressed in these studies is the lack of T cells specific for latency-associated antigens in active TB patients although it is likely that most TB patients undergo a phase of latent infection prior to active disease. As an initial attempt to address this point we determined T-cell responses twice (prior to and under drug treatment) for a subgroup of TB patients (data not shown) and detected increased frequencies for some of the latency proteins in patients under drug treatment. Therefore the lack of latency-associated T cells at least for some antigens seems to be restricted to the acute TB phase. This could be explained i) by the recruitment of these T cells to the lung or ii) increased T-cell regulation during the acute TB phase.

In contrast to intracellular IFNγ analyses, some IFNγ was also detected in supernatants of PBMC from a subgroup of TST-negative donors after stimulation with latency-associated antigens as described previously [5]. The second round of restimulation did not cause this higher background in TST-negative donors, since supernatants collected at day 6 from a subgroup of uninfected donors showed similar IFNγ abundance as compared to the day 7 time point after restimulation (data not shown). Using flow cytometry, we excluded the possibility that other immune cell populations (e.g. NK cells, NKT cells, CD8^+^ T cells) were the source of IFNγ (data not shown). A possible explanation would be exhaustion or induced cell death (e.g. by apoptosis) after restimulation 16 h prior to analysis [25]. Alternatively, these donors could be false negative in the T-Spot TB© although we have no evidence to support this assumption, or these responses reflect cross-reactivity towards antigens expressed by commensal bacteria [26].

In an initial attempt to characterize the T-cell responses against latency-associated antigens we compared the T-cell responses against immunodominant and latency-associated antigens from individual donors. While immunodominant antigens ESAT6_CFP-10, TB10.4, Ag85a induced significantly stronger T-cell responses in the 16-h short-term assay as compared to latency-associated antigens (i.e. Rv0569, Rv1734, Rv2003), comparable proportions of IFNγ-producing T cells were detected in the 7-day two rounds of restimulation assay. We conclude that a simply ‘boosted’ T-cell response by prolonged stimulation and two rounds of restimulation did not cause this effect as this would lead to higher proportions of T cells specific for immunodominant antigens. Distinct maturation stage of T-cell populations specific for latency-associated antigens may account for these findings. This may lead to differences in the activation threshold prerequisite for *in vitro* cytokine expression. Previous studies demonstrated higher sensitivity of a 6-day whole blood assay for T-cell responses in LTBI suggesting that prolonged *in vitro* restimulation preferentially induced central memory T cells [14], [16]. This supports the notion that T cells specific for latency-associated antigen have a distinct maturation/activation status. Detailed investigation of both T-cell populations will be performed in the future, including global gene expression profiling.

## Materials and Methods

### Ethics Statement

The clinical investigations have been conducted according to the principles expressed in the Declaration of Helsinki. All donors gave written informed consent and the local ethics committee of the University Hospital Berlin at Charité approved this study (205-18.1; 205-18.2; 205-18.3).

### Human subjects

Peripheral blood (40 ml) was obtained from 22 LTBI and 20 patients with active pulmonary TB. LTBI and TB patients were recruited at HELIOS clinic Emil-von-Behring and the Charité, both in Berlin; and at the Asklepios Clinic, Munich-Gauting. Diagnosis of LTBI was based on positive TST (>10 mm) and positive T-Spot TB™. TB diagnosis was based on patient history, chest X-ray, TST, and mycobacterial culture. Ten *M. tuberculosis* non-infected (TST-negative) donors were recruited among volunteers of the Max Planck Institute for Infection Biology, Berlin. All donors were Caucasians. There was no gender bias and only minor age differences between both study groups (Table 4).

**Table 4:** Characteristics of LTBI, TB patients and non-infected donors.

	LTBI^1^	TB patients^2^	Non-infected donors^3^
Total number	22	20	10
Female	11	10	5
Male	11	10	5
Age range years (median)	28–64 (47)	26–63 (41)	24–55 (36)

1 healthy latently *M. tuberculosis*-infected donors.

2 patients with active pulmonary TB.

3 healthy tuberculin skin test (TST)-negative donors.

### Recombinant M. tuberculosis proteins and synthetic peptide pools

Recombinant *M. tuberculosis* proteins (Table 1) were expressed in *Escherichia coli* and purified as described previously [5], [27]. Synthetic 15-mer peptide pools representing Rv3407 were generated with an overlap of 12 amino acids using combinatory chemistry (Jerini). The pools were designed as matrix pools as described before [28]. The peptides are listed in Table 3.

### Cell culture assays

Peripheral blood mononuclear cells (PBMC) were isolated by density centrifugation (Biocoll, Biochrom) following manufacturer's instructions and 2×10^5^ cells were cultured in 200 µL medium A [(RPMI-1640 (GIBCO, Invitrogen) with 10% human serum (Sigma-Aldrich), 100 U/ml penicillin, 100 µg/ml streptomycin, 1 mM L-glutamine and 10 mM HEPES (all PAA laboratories)] using 96-well round-bottom plates (NUNC). Each well was surrounded by wells filled with sterile water (200 µl) to avoid drying effects. In the short-term assay we stimulated PBMC for 16 h with different recombinant *M. tuberculosis* proteins (5 µg/ml, see Table 1), purified protein derivative (PPD) of *M. tuberculosis* (5 µg/ml) (Statens Serum Institute), Staphylococcus enterotoxin B (SEB) (1 µg/ml) (Sigma-Aldrich), and synthetic peptide pools (5 µg/ml per peptide) at 37°C and 5% CO_2_. After 4 h of incubation brefeldin A (10 µg/ml) (Sigma-Aldrich) was added.

In the long-term assay the same stimuli were applied at the beginning except for SEB that was added on day 6 to a well of non-stimulated cultured cells. For each of the other stimuli the same antigen was added on day 6 in 20 µl medium A using the same final concentrations. In the long-term assay, brefeldin A (10 µg/ml) was added 4 h after the second restimulation only, and cells were then cultured for an additional 12 h.

**Figure 5:**
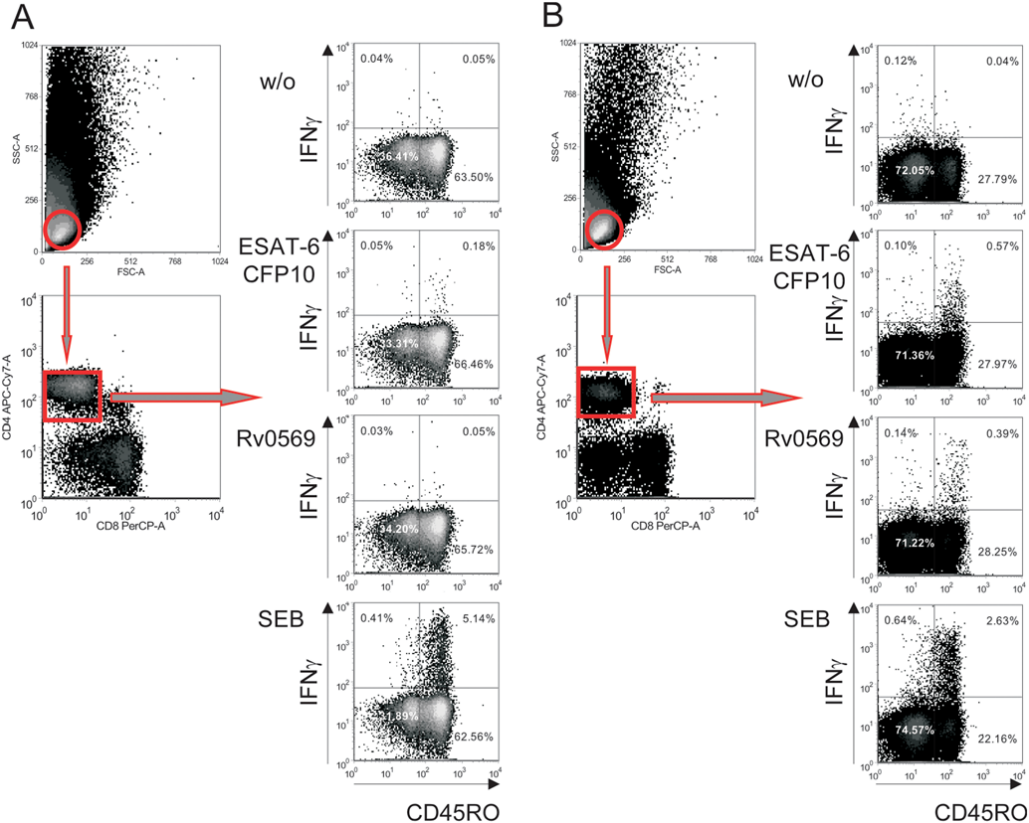
Gating procedures of flow cytometry analyses to determine protein candidate specific T cell proportions. Representative analyses from a patient with Tb (A) and an LTBI (B) are shown. Open red circles and dot plot connected by red arrows indicate the sequence of analysis steps. First, lymphocytes were gated using size (forward scatter; FSC) and granularity (side scatter, SSC). These cells were then analyzed for CD4 expression. CD4^+^ T cells were analyzed for IFNγ CD45RO expression for each stimulation (without stimulus, w/o; proteine 3; protein 11; SEB). Proportions of CD45RO_high_ IFNγ expressing CD4^+^ T cells (upper right quadrants) were determined. The background of non-stimulated T cells (w/o) was subtracted for analyses.

Afterwards, for both assays, cells were fixed and permeabilized using cytofix/cytoperm™ (BD Biosciences) following manufacturer's instructions and stained with the following fluorochrome-labeled monoclonal antibodies: α-CD3 (Pacific Blue) (BioLegend), α-CD4 (APC-Cy7), α-CD8 (PerCP), α-CD45RO (Pe-Cy7), α-IFNγ (APC) (all BD Biosciences). After staining for 45 min at 4°C, cells were washed twice in cytoperm/wash, and once in PBS containing 10% FCS. Measurements and analyses were performed using a LSRII flow cytometer and FACS-Diva software (both BD Biosciences). Examples of the procedures of analysis are shown in Figure 5A for a TB patient and Figure 5B for an LTBI. About 18000 CD4^+^ CD45RO^+^ T cells were collected for each sample. For background determinations, values of non-stimulated controls were subtracted from the different stimuli of each individual donor.

### Cytokine analyses in culture supernatants by ELISA

PBMC (5×10^4^) were added to 96-well round-bottom plates (NUNC) in 200 µL medium. Cells were stimulated for 7 days as described in the previous section but without adding brefeldin A. We harvested 110 µl of cell culture supernatant at day 7. The IFNγ ELISA (BD Biosciences) was performed according to manufacturer's guidelines. Plates were analyzed by measuring extinction at 450 nm using an ELISA plate reader (Molecular Devices).

### Donor classification and significance analyses

The discriminatory power for classifying TB patients and LTBI was investigated using random forest analysis [29] based on the proportion of IFNγ-expressing CD4^+^, CD45RO^+^ T cells against 13 selected stimuli (i.e. PPD, ESAT6_CFP10, Rv0569, Rv1733c, Rv1734, Rv2003, Rv2005c, Rv2006, Rv0140, Rv1009, Rv1884c, Rv2450c, and Rv3407). We determined the relative feature importance for discrimination using a leave-1-out cross validation for all possible combinations of genes and assessed the proportion of correctly classified patients in the left-out group.

For significance analyses the Mann-Whitney U-test was used. Significance of two-sided p-values is indicated as follows: * *P*<0.05, ** *P*<0.01, and *** *P*<0.001.
